# Association Between Increased Central and Peripheral Arterial 2 Stiffness and Vitamin Intake in Healthy Adults: EVA Follow-Up 3 Study

**DOI:** 10.3390/nu18050745

**Published:** 2026-02-26

**Authors:** Javier Alonso-Diaz, Marta Gómez-Sánchez, Andrea Sánchez-Moreno, Cristina Lugones-Sánchez, Emiliano Rodriguez-Sanchez, Luis Garcia-Ortiz, Leticia Gómez-Sánchez, Manuel A. Gómez-Marcos

**Affiliations:** 1 Primary Care Research Unit of Salamanca (APISAL), Avd. Portugal, 37005 Salamanca, Spain; jalonsodiaz@usal.es (J.A.-D.); martagmzsnchz@gmail.com (M.G.-S.); andreasan@usal.es (A.S.-M.); cristinals@usal.es (C.L.-S.); emiliano@usal.es (E.R.-S.); lgarciao@usal.es (L.G.-O.); leticiagmzsnchz@gmail.com (L.G.-S.); 2 Institute of Biomedical Research of Salamanca (IBSAL), Paseo de San Vicente, 37007 Salamanca, Spain; 3 Home Hospitalization Service, Marqués of Valdecilla University Hospital, 39008 Santander, Spain; 4 Research Network on Chronicity, Primary Care and Health Promotion (RICAPPS), 37005 Salamanca, Spain; 5 Faculty of Nursing and Physiotherapy, University of Salamanca, Avenida de los Donantes de Sangre, s/n, 37008 Salamanca, Spain; 6 Salamanca Primary Care Management, Castilla and León Health Service–SACYL, 37005 Salamanca, Spain; 7 Department of Medicine, University of Salamanca, Av. Campo Charro, 37007 Salamanca, Spain; 8 Department of Biomedical and Diagnostic Sciences, University of Salamanca, Av. Campo Charro, 37007 Salamanca, Spain; 9 Emergency Service, University Hospital of La Paz. P. of Castellana, 261, 28046 Madrid, Spain

**Keywords:** arterial stiffness, longitudinal study, healthy adults, carotid–femoral pulse wave velocity, brachial–ankle pulse wave velocity, dietary vitamins

## Abstract

Background: Evidence from prospective studies on the relationship of the dietary vitamin intake and the progression of central and peripheral arterial stiffness remains limited. Objective: To evaluate the association between dietary vitamin intake with the changes in central and peripheral arterial stiffness over a five-year follow-up in adults without previous cardiovascular disease. Methods: This five-year longitudinal study included 466 participants from the EVA study who were evaluated at baseline and follow-up (mean age 55.96 ± 14.15 years; 51.1% women). Central arterial stiffness was assessed using carotid–femoral pulse wave velocity (cfPWV), and peripheral arterial stiffness was measured using brachial–ankle pulse wave velocity (baPWV). Dietary vitamin intake was estimated using the EVIDENT smartphone application, developed and validated by CGB and the Salamanca Primary Care (APISAL; registration number 00/2014/2207). Results: In multivariable linear regression analyses adjusted for age, sex, lifestyle factors, and cardiovascular risk factors, greater increases in cfPWV were inversely associated with vitamin B9 (folate) intake (β = −0.233; 95% CI: −0.390 to −0.075) and vitamin C intake (β = −0.291; 95% CI: −0.507 to −0.075). Similarly, increases in baPWV were inversely associated with vitamin B9 intake (β = −0.156; 95% CI: −0.287 to −0.025) and vitamin C intake (β = −0.223; 95%CI: −0.402 to −0.044). Conclusions: The progression of central and peripheral arterial stiffness over five years was greater in individuals with lower dietary intakes of vitamin B9 and vitamin C. These findings provide novel evidence supporting the possible role of dietary vitamin intake in the progression of arterial stiffness with aging.

## 1. Introduction

Central arterial stiffness (AS), assessed by carotid femoral pulse wave velocity (cfPWV) [1], and peripheral arterial stiffness, measured by brachial–ankle pulse wave velocity (baPWV) [2], are independently associated with cardiovascular disease (CVD) morbidity and mortality, beyond traditional cardiovascular risk factors (CVRFs) [3,4]. Arterial stiffness is currently considered a robust biomarker of cardiovascular risk (CVR), as it allows the detection of structural and functional changes in the arterial wall before the onset of clinically overt CVD [2,5]. Moreover, several studies have shown that AS provides incremental prognostic information beyond traditional CVRFs [5], including blood pressure [1,5].

In recent decades, it has become increasingly evident that, together with the classic risk factors, nutritional status plays a relevant role important in the evaluation of vascular health. Beyond macronutrients, specific dietary components—micronutrients and vitamins—also appear to play a central role in the regulation of arterial stiffness [6,7].

Several vitamins are involved in key in metabolic processes of the organism such as oxidative stress control, nitric oxide bioavailability, homocysteine metabolism, and the regulation of vascular calcification, all of which are implicated in the pathophysiology of AS [7]. These interactions suggest that vitamin status may influence the mechanical properties of the arterial wall both directly and indirectly through multiple metabolic and cellular pathways.

Among fat-soluble vitamins, vitamin D has been one of the most extensively studied in relation to vascular function and AS. Observational studies have found an inverse association between circulating 25-hydroxyvitamin D concentrations and AS, assessed by cfPWV, in both the adult population in hypertensive, type 2 diabetics or kidney patients individuals [8]. A meta-analysis has shown correction of vitamin D deficiency with doses of at least 2000 international units (IU/day of vitamin D_3_ for more than four months was associated with modest reductions in pulse wave velocity (PWV) (standardized mean difference = −0.10;95% confidence interval (CI): −0.24 to 0.04; *p* = 0.17; *n* = 806 across ten studies) [9]. Additionally, a trial using high-dose monthly supplementation (100,000 IU of vitamin D3 for three months) in vitamin D–deficient adults reported a reduction in central augmentation index among participants with higher oxidative stress levels, although no overall improvement in PWV was observed [10]. Vitamin D may influence AS through multiple mechanisms, including modulation of the renin–angiotensin–aldosterone system, improvement of endothelial function and nitric oxide bioavailability, and reduction in inflammation and oxidative stress [11].

Antioxidant vitamins (C and E), have also been proposed as modulators of AS due to their ability to reduce vascular oxidative stress, preserve endothelial function, and improve nitric oxide bioavailability, a key mediator of arterial vasodilation [12]. Vitamin C contributes to vascular protection by reducing oxidative stress and enhancing nitric oxide bioavailability. In addition, other studies have shown that vitamin C supplementation may induce modest but significant reductions in AS, particularly in individuals with higher oxidative stress burden or CVRFs [13,14]. Vitamin E, mainly in the form of α-tocopherol, has been extensively studied for its role as a lipid-soluble antioxidant and its capacity to protect cell membranes from lipid peroxidation. In the vascular context, vitamin E contributes to the preservation of endothelial function by limiting oxidative damage and modulating inflammatory processes involved in arterial remodeling. Experimental and clinical studies suggest that vitamin E may improve nitric oxide bioavailability and reduce AS, particularly in populations with increased oxidative stress or CVR, although the effects appear to depend on baseline antioxidant status, dose, and intervention duration [15,16,17].

Carotenoids and vitamin A have also been linked to anti-inflammatory and antioxidant effects, although direct evidence regarding their specific impact on AS remains limited and largely indirect [7].

Finally, B-complex vitamins play a central role in homocysteine metabolism, an amino acid whose elevation has been associated with endothelial dysfunction and increased AS. In particular, vitamins B6, B9 (folate), and B12 are directly involved in homocysteine metabolism, and elevated homocysteine concentrations have been associated with higher AS values in different populations [18,19]. Hyperhomocysteinemia, which is common in older adults, has been associated with increased AS, and observational studies have reported correlations between elevated homocysteine levels, vitamin B9 or vitamin B12 deficiency, and vascular dysfunction [20]. However, although interventions with B-vitamin supplementation (such as the B-PROOF trial) successfully reduced homocysteine levels, they did not demonstrate significant improvements in PWV, suggesting a complex relationship potentially influenced by other factors [21]. Riboflavin (vitamin B2) has shown a decrease in blood pressure and AS in individuals with specific genetic variants, suggesting a possible indirect role in AS modulation [22,23]. Niacin (vitamin B3) has also demonstrated vascular benefits. In a study of healthy middle-aged and older adults, higher dietary niacin intake was associated with better indicators of endothelial function and lower markers of vascular oxidative stress, suggesting a protective effect on endothelial cells and arterial wall dynamics that may influence AS [24,25].

Overall, available evidence suggests that several vitamins may play a relevant role in modulating AS through multiple pathophysiological mechanisms. However, existing results are heterogeneous and largely derived from observational studies or short-term interventions. Therefore, further well-designed studies are needed to clarify the relationship between vitamin intake and AS and its potential role in CVR prevention.

Accordingly, the objective of this study was to evaluate the association between dietary vitamin intake and central (cfPWV) and peripheral (baPWV) arterial stiffness at baseline and over a five-year follow-up in adults without previous cardiovascular disease.

## 2. Materials and Methods

### 2.1. Study Design, Participants and Sample Size

A longitudinal study was conducted on participants from the Association between different risk factors and vascular accelerated ageing study (EVA study; NCT02623894) [26]. The study population consisted of individuals receiving healthcare in five urban primary care centers. Using stratified random sampling with replacement by age group (35, 45, 55, 65, and 75 years) and sex, 501 participants were selected (approximately 100 per age group, 50% women), from a reference population of 43,946 individuals.

Baseline evaluation and inclusion were conducted between June 2016 and November 2017, and follow-up assessment took place between May 2021 and October 2022. Inclusion criteria were age between 35 and 75 years and provision of written informed consent. Exclusion criteria included terminal illness, inability to attend healthcare centers, history of cardiovascular disease, estimated glomerular filtration rate < 30 mL/min/1.73 m^2^, chronic inflammatory disease or acute inflammatory process within the previous three months, or treatment with estrogens, testosterone, or growth hormone.

At the five-year follow-up, 480 participants were evaluated. The final analytical sample consisted of 466 individuals who completed both assessments and had complete three-day dietary intake records. Flowcharts describing baseline evaluation and five-year losses can be seen in the Figures S1 and S2. This work follows the recommendations outlined in the STROBE guidelines [27].

### 2.2. Ethical Considerations

The study was approved by the Committee of Ethics of Research with Medicines of the Health Area of Salamanca on 4 May 2015 (baseline study) and 13 November 2020 (follow-up study; reference code PI 2020 10 569, approval date: 13 November 2020). Before being included in the study, the subjects signed consent forms and the data collection followed the Declaration of Helsinki [28] and World Health Organization (WHO) guidelines for observational studies. Data were anonymized and coded using unique alphanumeric identifiers prior to analysis, and access to personal data was restricted to authorized research personnel.

### 2.3. Dependent Variables

Central arterial stiffness was assessed using cfPWV with the SphygmoCor device (AtCor Medical Pty Ltd., West Ryde, Australia). Pulse waves were recorded at the carotid and femoral arteries with participants in the supine position. Transit time was estimated relative to the electrocardiogram (ECG) R wave, and cfPWV was calculated based on measured distances from the suprasternal notch to the carotid and femoral recording sites using a measuring tape [1,29].

Peripheral arterial stiffness was assessed using baPWV with the VaSera VS-1500 device (Fukuda Denshi Co., Ltd., Tokyo, Japan), following manufacturer instructions. Cuffs were placed on both arms and ankles, and a phonocardiographic microphone was positioned at the second intercostal space. baPWV was calculated using the equation: baPWV = (0.5934 × height (cm) + 14.4724)/tba, where tba is the time interval between brachial and ankle pulse waves [2,30].

Participants were instructed to avoid caffeine, smoking, and exercise for at least three hours prior to measurements and to rest quietly for five minutes before assessment.

### 2.4. Independent Variables

Dietary vitamin intake was assessed using a three-day food record collected via the EVIDENT smartphone application. The EVIDENT app was developed and validated by CGB and the Primary Care Research Group of Castilla y León (REDIAPP), with intellectual property registration number 00/2014/2207 [31]. Foods were organized into predefined groups, and participants recorded all daily meals. Based on food composition tables and portion sizes, intake of vitamins A, B1, B2, B3, B6, B9, B12, and C was estimated. Vitamin D was determined through laboratory analysis.

Figure S3 shows EVIDENT app main screen and selection of dishes.

### 2.5. Potential Confounding Variables

Age and sex were recorded. Lifestyle factors included smoking status, alcohol consumption (g/week), adherence to the Mediterranean diet assessed by the 14-item Mediterranean Diet Adherence Screener (MEDAS) questionnaire [32], sedentary behavior assessed by the Marshall Sitting Questionnaire [33,34], and physical activity evaluated using the International Physical Activity Questionnaire–Short Form (IPAQ-SF) [35,36], expressed as metabolic equivalents of task (MET)-min/week.

Fasting blood samples were used to assess lipid profile and fasting glucose. Blood pressure and heart rate were measured using a validated OMRON M10-IT sphygmomanometer, following European Society of Hypertension recommendations [37]. Pulse pressure was calculated as systolic minus diastolic blood pressure. Body weight and height were measured using standardized procedures, and body mass index (BMI) was calculated.

### 2.6. Statistical Analysis

Continuous variables are presented as mean ± standard deviation, and categorical variables as number and percentage. Correlations between baseline and five-year changes in arterial stiffness and vitamin intake were assessed using Spearman’s correlation coefficient. Multivariable linear regression models were used to evaluate associations between arterial stiffness (baseline values and five-year changes) and vitamin intake, adjusting for age, sex, lifestyle factors, and cardiovascular risk factors.

All analyses were performed using IBM SPSS Statistics version 28 (IBM Corp., Chicago, IL, USA), and *p* < 0.05 was considered statistically significant.

## 3. Results

### 3.1. Characteristics of the Participants Included in the Baseline Evaluation and with Five-Year Follow-Up and Recorded Vitamin Intake

A total of 466 participants with available baseline and five-year measurements of central and peripheral arterial stiffness and complete vitamin intake records obtained through the EVIDENT app were included in the analysis. The mean age was 56.00 ± 14.20 years, and 50.6% were women.

Baseline characteristics of the study population, overall and stratified by sex, are presented in Table 1. Men reported higher levels of physical activity, longer sedentary time, and greater alcohol consumption than women. In addition, men showed higher values of blood pressure, body weight, height, BMI, and cfPWV compared with women. Conversely, women exhibited a higher Mediterranean diet adherence score than men.

Overall, the five-year increase in cfPWV was 1.14 ± 1.75 m/s, and the increase in baPWV was 0.90 ± 1.53 m/s. When stratified by sex, the increase in cfPWV was significantly greater in men than in women (1.32 ± 1.75 vs. 0.96 ± 1.75 m/s; *p* = 0.023). In contrast, the increase in baPWV was lower in men than in women (0.78 ± 1.55 vs. 1.02 ± 1.51 m/s; *p* = 0.045).

Vitamin intake assessed using the EVIDENT app is shown in Table 2, overall and by sex. Men had a higher intake of vitamin B3 than women (*p* = 0.006), while no significant sex differences were observed for the remaining vitamins analyzed.

### 3.2. Relationship Between Baseline Central and Peripheral Arterial Stiffness and Their Five-Year Changes

**Figure 1:**
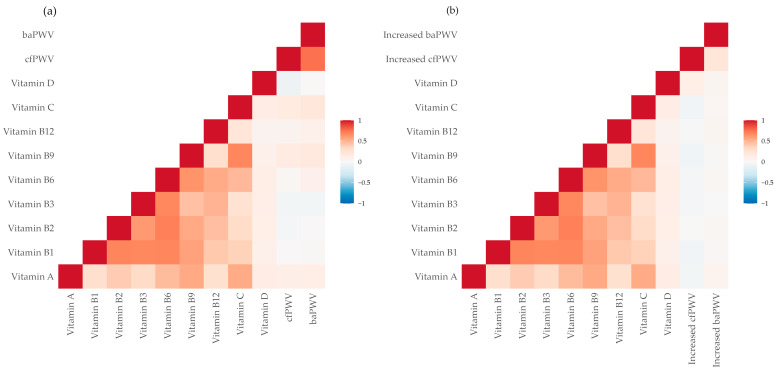
Correlation between dietary vitamin intake and central and peripheral arterial stiffness at baseline (**a**) and with five-year changes in arterial stiffness (**b**).

Figure 1 shows the correlations between baseline central and peripheral arterial stiffness (panel a) and their five-year changes (panel b) with dietary vitamin intake.

### 3.3. Association Between Baseline and Five-Year Changes in Central and Peripheral Arterial Stiffness After Multivariable Adjustment

**Figure 2:**
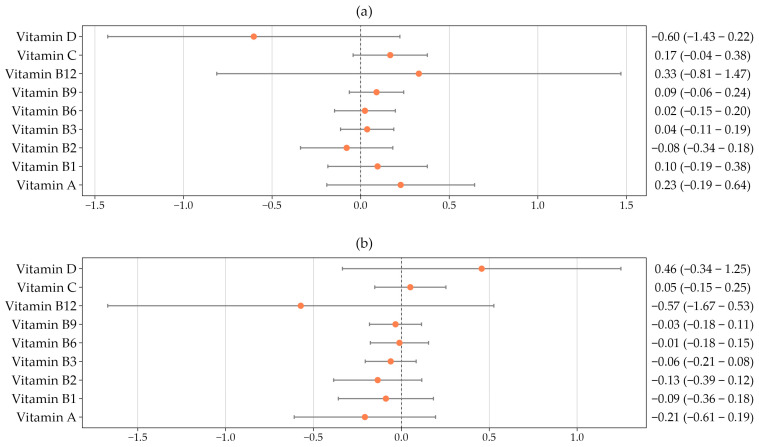
Association between dietary vitamin intake and baseline central arterial stiffness (**a**) and baseline peripheral arterial stiffness (**b**), adjusted for age, sex, lifestyle factors, and cardiovascular risk factors.

Figure 2 illustrates the associations between baseline central (panel a) and peripheral (panel b) arterial stiffness and dietary vitamin intake after adjustment for age, sex, lifestyle factors, and cardiovascular risk factors. No significant associations were observed between vitamin intake and baseline measures of arterial stiffness.

**Figure 3:**
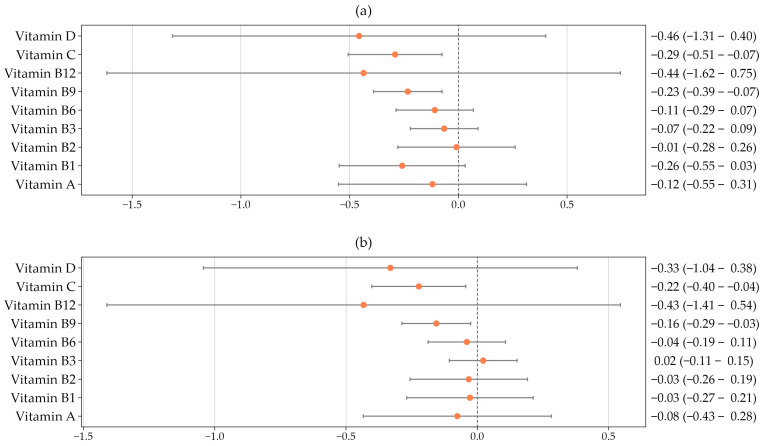
Association between dietary vitamin intake and five-year changes in central arterial stiffness (**a**) and peripheral arterial stiffness (**b**), adjusted for age, sex, lifestyle factors, and cardiovascular risk factors.

Figure 3 presents the associations between changes in central (panel a) and peripheral (panel b) arterial stiffness over five years and dietary vitamin intake, after adjustment for age, sex, lifestyle factors, and cardiovascular risk factors. A greater increase in cfPWV was inversely associated with vitamin B9 intake (β = −0.233; 95% CI: −0.390 to −0.075) and vitamin C intake (β = −0.291; 95% CI: −0.507 to −0.075). Similarly, the increase in baPWV was inversely associated with vitamin B9 intake (β = −0.156; 95% CI: −0.287 to −0.025) and vitamin C intake (β = −0.223; 95% CI: −0.402 to −0.044).

## 4. Discussion

### 4.1. Main Findings

In this longitudinal study conducted within the framework of the EVA study, we examined the association between dietary vitamin intake and central and peripheral arterial stiffness, both at baseline and over a five-year follow-up period. The main findings indicate that higher dietary intakes of vitamin B9 (folate) and vitamin C were independently associated with a lower progression of arterial stiffness, as assessed by cfPWV and baPWV, after adjustment for age, sex, lifestyle factors, and cardiovascular risk factors. In contrast, no significant associations were observed with baseline arterial stiffness measures.

These results suggest that dietary vitamin intake may be more strongly related to the progression of arterial stiffening over time rather than to cross-sectional differences in arterial stiffness.

### 4.2. Sex Differences in Arterial Stiffness

In the present study, the increase in cfPWV over five years was greater in men than in women, whereas the increase in baPWV was higher in women. There is substantial evidence indicating that the mechanisms underlying arterial stiffening differ between men and women. Experimental and clinical studies have shown that women follow a distinct trajectory of arterial stiffness across the life course, with a more pronounced acceleration after menopause, suggesting a relevant role of hormonal factors [38].

In addition, women have been reported to be more susceptible to increases in arterial stiffness associated with higher body mass index [39]. Sex-related differences in arterial stiffness progression before and after puberty have also been described, further supporting the existence of distinct hemodynamic and structural mechanisms between men and women [40]. These differences may partly explain the divergent patterns observed for central and peripheral arterial stiffness in our study.

### 4.3. Sex Differences in Dietary Intake

In the study population, dietary intake of vitamin B3 was higher in men than in women, whereas no significant sex differences were observed for the remaining vitamins. Conversely, women showed a higher Mediterranean diet adherence score than men. These findings are partially consistent with previous reports in the literature [41].

Within the EVA study, sex differences have previously been observed in the intake of several vitamins, including vitamins A, C, vitamin B9, and vitamin B12, as well as in their associations with vascular function parameters [42]. This suggests that dietary vitamin intake patterns may reflect different nutritional exposures between men and women, which could influence vascular ageing trajectories.

Consistently, studies conducted in Mediterranean populations have reported higher plasma folate concentrations in women than in men, despite variable dietary intake [43]. In contrast, a higher prevalence of vitamin D deficiency has been reported among women, which may contribute to sex-related differences in vascular health and cardiovascular risk [44]. Overall, these findings highlight the importance of considering sex as a relevant effect modifier when evaluating associations between vitamin intake and arterial stiffness.

### 4.4. Vitamin D

In the present study, no significant associations were observed between arterial stiffness measures and vitamin D intake. In the literature, numerous observational studies have reported inverse associations between circulating 25-hydroxyvitamin D concentrations and central arterial stiffness assessed by cfPWV, both in the general population and in individuals with cardiovascular risk factors [45,46]. Similar associations have been reported for peripheral arteries, suggesting a systemic vascular effect of vitamin D [46].

Proposed mechanisms include modulation of the renin–angiotensin–aldosterone system, reduction in vascular inflammation, improvement of endothelial function, and inhibition of vascular smooth muscle cell proliferation [47]. However, randomized controlled trials of vitamin D supplementation have yielded inconsistent results, with several studies failing to demonstrate significant reductions in arterial stiffness, particularly in individuals without baseline vitamin D deficiency or with limited treatment duration [48,49].

The absence of an association in the present study may be related to differences in assessment methods, characteristics of the study population, age distribution, or geographic and lifestyle factors.

### 4.5. Antioxidant Vitamins: Vitamin C and Contextual Evidence on Vitamin A

In the present study, only dietary vitamin C intake was independently associated with a lower progression of central and peripheral arterial stiffness over the five-year follow-up period. No significant associations were observed between vitamin A intake and arterial stiffness parameters. For this reason, vitamin A is discussed solely within a general pathophysiological context related to the role of antioxidant vitamins in vascular health.

Vitamin C plays a key role in vascular protection by reducing oxidative stress, improving endothelial function, and preserving nitric oxide bioavailability. Consistent with our findings, several clinical trials and meta-analyses have shown that vitamin C supplementation is associated with modest but significant reductions in arterial stiffness and improvements in endothelial function, particularly in populations with a higher oxidative stress burden or cardiovascular risk factors [13,14].

In addition, vitamin C contributes to vascular antioxidant defense by regenerating oxidized vitamin E, thereby preserving its lipid-phase antioxidant activity in cell membranes, which may further enhance protection against oxidative stress–induced arterial remodeling [16,17].

Vitamin A and related carotenoids also exhibit antioxidant and anti-inflammatory properties that could indirectly contribute to vascular protection. However, the available evidence regarding their specific impact on arterial stiffness is limited and inconsistent and is mainly derived from experimental or observational studies. In line with this literature, no significant associations were identified between vitamin A intake and central or peripheral arterial stiffness in our study.

Overall, these results suggest that, within the group of antioxidant vitamins, vitamin C may play a more relevant role in modulating the progression of arterial stiffness than other vitamins with antioxidant properties, such as vitamin A.

Our findings demonstrate an inverse association between arterial stiffness progression and dietary vitamin C intake. This is consistent with clinical trials reporting that vitamin C supplementation may improve endothelial function and reduce arterial stiffness, particularly in individuals with hypertension or increased oxidative stress [50]. In line with this, a systematic review and subsequent meta-analyses have reported modest but significant reductions in PWV following antioxidant vitamin supplementation [42,43].

The magnitude of these effects appears to depend on baseline oxidative stress and nutritional status [51]. These observations are biologically plausible, given the central role of oxidative stress in the pathophysiology of arterial stiffness, including elastin degradation, increased collagen deposition, and reduced nitric oxide bioavailability. In this context, antioxidant vitamins such as vitamin C and vitamin A may exert protective effects on the arterial wall [51,52].

Excessive production of reactive oxygen and nitrogen species (ROS/RNS), mainly derived from mitochondrial respiration, NADPH oxidase activity, uncoupled endothelial nitric oxide synthase, and inflammatory pathways, plays a central role in vascular ageing. Sustained oxidative stress promotes endothelial dysfunction and arterial remodeling through activation of redox-sensitive signaling pathways involved in apoptosis, necrosis, and cell death pathways. In this context, vitamin C may limit ROS/RNS accumulation through direct free radical scavenging, regeneration of oxidized antioxidants such as vitamin E, and preservation of nitric oxide bioavailability. In parallel, adequate vitamin B9 intake may indirectly reduce oxidative stress by modulating homocysteine metabolism and preventing homocysteine-induced endothelial damage, thereby contributing to vascular protection [12,13,18,19,20,21,53].

### 4.6. B-Complex Vitamins

An inverse association was observed between arterial stiffness progression and dietary intake of vitamin B9. Vitamin B9 and B12 play a key role in homocysteine metabolism, and elevated homocysteine levels have been associated with endothelial dysfunction, vascular inflammation, and increased arterial stiffness [54,55]. Our findings are consistent with observational studies reporting inverse associations between vitamin B9 or vitamin B12 status and arterial stiffness measures [53].

However, intervention studies have produced less consistent results. In the B-PROOF trial, supplementation with folic acid and vitamin B12 significantly reduced homocysteine concentrations but did not lead to significant improvements in arterial stiffness assessed by PWV [56,57]. This suggests that the relationship between B-vitamin intake, homocysteine metabolism, and arterial stiffness is complex and may depend on additional factors such as baseline nutritional status, age, and duration of exposure.

### 4.7. Clinical Implications

The results of this study suggest that optimizing dietary intake of specific vitamins, particularly vitamins B9 and C, may represent an accessible and low-risk strategy to limit the progression of arterial stiffness in healthy adults. This may have implications for long-term cardiovascular risk reduction, especially in populations with suboptimal dietary patterns.

While the magnitude of the observed associations was modest, it should be interpreted in the context of the natural progression of arterial stiffness with ageing. In our cohort, the average five-year increase in cfPWV was approximately 1.1 m/s, a change that has been associated with clinically relevant increases in cardiovascular risk. Therefore, even relatively small differences in the progression of arterial stiffness associated with higher dietary intake of vitamin C and vitamin B9 may be meaningful, particularly when sustained over long periods. From a population perspective, modest shifts in arterial stiffness progression may contribute to a lower burden of vascular ageing and cardiovascular risk, beyond mere statistical significance.

Although multivariable models were adjusted for adherence to the Mediterranean diet using the MEDAS score, residual confounding by overall diet quality or dietary patterns cannot be completely ruled out. Dietary patterns reflect complex combinations of foods and nutrients that may not be fully captured by individual nutrient analyses or summary adherence scores. Therefore, the associations observed between vitamin C and vitamin B9 intake and arterial stiffness progression should be interpreted within the context of overall dietary habits rather than as isolated nutrient effects.

From a nutritional perspective, vitamin C is mainly obtained from fruits and vegetables, with citrus fruits (e.g., oranges providing approximately 50–70 mg per medium fruit), kiwifruit (≈70–90 mg per unit), strawberries (≈60 mg per 150 g), and bell peppers (≈80–120 mg per 100 g) representing major dietary sources. Vitamin B9 is primarily supplied by green leafy vegetables such as spinach (≈140 µg per 100 g), legumes including lentils (≈180 µg per cooked cup), and chickpeas (≈280 µg per cooked cup), as well as by whole grains and fortified cereals. Vitamin A intake is mainly derived from retinol in animal-based foods, such as liver (≈6500–9000 µg retinol equivalents per 100 g), and from provitamin A carotenoids in plant foods, including carrots (≈800–900 µg retinol equivalents per 100 g) and leafy green vegetables [58,59].

From a clinical perspective, our findings suggest that dietary vitamin intake, particularly vitamin C and vitamin B9, may be associated with the progression of arterial stiffness over time. However, given the observational design of this study, causality cannot be inferred. These results should therefore be interpreted as hypothesis-generating and indicative of potential associations within the context of overall dietary habits. Further prospective studies and randomized controlled trials are needed to determine whether targeted dietary interventions or optimization of vitamin intake can effectively modify arterial stiffness progression and reduce vascular ageing risk.

### 4.8. Limitations and Strengths

Several limitations should be acknowledged. First, although the sample size was adequate, a larger population may have allowed the detection of additional associations [60]. Second, the study was conducted in a specific Spanish cohort, which may limit the generalizability of the findings to other populations [61]. Third, dietary vitamin intake was assessed using self-reported records, which may be subject to measurement error [62]. Finally, social determinants that influence dietary intake, as highlighted by the World Health Organization, were not included in the analyses [63].

Dietary vitamin intake was assessed using three-day self-reported food records collected through a smartphone application, which may be subject to reporting bias, random measurement error, and within-person variability in micronutrient intake. Although the EVIDENT application has been previously validated and allows detailed recording of food consumption, short-term dietary records may not fully capture habitual intake, particularly over a long follow-up period. In this context, day-to-day variability in vitamin consumption could have led to exposure misclassification. However, such non-differential measurement error would be expected to attenuate observed associations, potentially underestimating the true relationship between dietary vitamin intake and the progression of arterial stiffness. A major strength of this study is its longitudinal design with a five-year follow-up and the inclusion of 466 participants selected through age- and sex-stratified random sampling from a large reference population.

## 5. Conclusions

In this five-year longitudinal study including 466 adults without previous cardiovascular disease, higher dietary intakes of vitamin C and vitamin B9 (folate) were independently associated with a lower progression of both central and peripheral arterial stiffness, assessed by carotid–femoral and brachial–ankle pulse wave velocity. These associations were observed after adjustment for age, sex, lifestyle factors, and cardiovascular risk factors, and were evident for arterial stiffness progression rather than for baseline measurements.

Given that arterial stiffness is a robust early marker of vascular ageing and cardiovascular risk, our findings suggest that suboptimal intake of specific vitamins may contribute to accelerated arterial stiffening over time. From a broader perspective, these results support the potential relevance of adequate dietary intake of vitamin C and vitamin B9 as part of healthy dietary patterns aimed at preserving vascular health during ageing. Further prospective studies and intervention trials are warranted to confirm these associations and to clarify their implications for cardiovascular prevention strategies.

## Figures and Tables

**Table 1:** Baseline characteristics of the subjects included in the EVA study and who Performed follow-up at 5 years and consumption record of vitamins overall and by sex.

Variable	Global (n = 466)	Men (n = 226)	Women (n = 240)	*p* Value
Age (years)	55.96 ± 14.15	55.94 ± 14.19	55.98 ± 14.13	0.973
Alcohol intake (gr/sem)	44.89 ± 75.95	50.71 ± 93.90	20.57 ± 43.49	<0.001
Smoking status, n (%)	82 (17.6)	43 (9.2)	39 (8.4)	0.253
Mediterranean Diet Score	7.17 ± 2.07	6.73 ± 1.98	7.58 ± 2.08	<0.001
Physical activity total (Mets/min/week)	2537 ± 3327	3321 ± 3835	1798 ± 2562	<0.001
Sitting hours (hours/week)	42.14 ± 17.80	47.97 ± 16.54	36.66 ± 17.23	<0.001
Systolic blood pressure (mmHg)	119.70 ± 17.76	125.73 ± 16.49	114.03 ± 17.06	<0.001
Diastolic blood pressure (mmHg)	75.64 ± 10.00	77.68 ± 9.15	73.73 ± 10.39	<0.001
Pulse pressure (mmHg)	44.06 ± 12.32	48.05 ± 12.03	40.30 ± 11.40	<0.001
Resting heart rate (beats/min)	68.72 ± 9.52	67.65 ± 9.99	69.73 ± 8.96	0.018
Fasting glucose (mg/dL)	87.91 ± 16.71	89.97 ± 18.58	85.98 ± 14.51	0.010
Total cholesterol (mg/dL)	194.97 ± 32.84	192.61 ± 33.08	197.20 ± 32.52	0.132
LDL cholesterol (mg/dL)	115.58 ± 29.51	117.60 ± 30.57	113.68 ± 28.42	0.153
Weight (kg)	72.48 ± 13.85	79.73 ± 11.85	65.65 ± 12.02	<0.001
Height (cm)	165.06 ± 9.69	171.81 ± 7.26	158.70 ± 7.02	<0.001
Body mass index (kg/m^2^)	26.55 ± 4.22	26.98 ± 3.41	26.13 ± 4.82	0.027
cfPWV (m/s)	8.21 ± 2.56	8.66 ± 2.78	7.79 ± 2.25	<0.001
baPWV (m/s)	12.91 ± 2.65	13.15 ± 2.45	12.68 ± 2.80	0.058

Categorical variables shown as n (%). Continuous variables shown as mean ± standard deviation. *p* value: Differences between the sexes. cfPWV: Femoral carotid pulse wave velocity; baPWV: Brachial ankle pulse wave velocity.

**Table 2:** Vitamin intake according to the EVIDENT study app record overall and by sex.

Variable	Global (n = 466)	Men (n = 226)	Women (n = 240)	*p* Value
Vitamin A (mcg/day)	5598 ± 3842	5398 ± 3766	5787 ± 3910	0.274
Vitamin B1 (mg/day)	1.65 ± 0.58	1.70 ± 0.58	1.60 ± 0.57	0.067
Vitamin B2 (mg/day)	1.87 ± 0.62	1.90 ± 0.59	1.85 ± 0.65	0.433
Vitamin B3 (mg/day)	38.72 ± 10.83	40.13 ± 10.56	37.39 ± 10.94	0.006
Vitamin B6 (mg/day)	2.63 ± 0.95	2.66 ± 0.93	2.58 ± 0.97	0.360
Vitamin B9 (folate) (mg/day)	329 ± 111	321 ± 102	336 ± 119	0.141
Vitamin B12 (mg/day)	12.26 ± 13.90	13.35 ± 14.92	11.23 ± 12.81	0.100
Vitamin C (mg/day)	176 ± 81	169 ± 83	182 ± 79	0.084
Vitamin D (mcg/day)	25.54 ± 19.73	24.49 ± 10.02	26.59 ± 26.06	0.265

Note: Categorical variables shown as n (%). Continuous variables shown as mean ± standard deviation. *p* value: Differences between the sexes.

## Data Availability

The variables that we use in the analyses carried out to obtain the results of this work are available upon reasonable request to the corresponding author.

## Supplementary Materials

The following supporting information can be downloaded at: https://www.mdpi.com/article/10.3390/nu18050745/s1, Figure S1, EVA study flowchart; Figure S2, flowchart of the 5-year follow-up phase; Figure S3, EVIDENT app main screen and selection of dishes.

## Abbreviations

The following abbreviations are used in this manuscript:

APISAL	Primary Care Research Unit of Salamanca
AS	Arterial stiffness
baPWV	Brachial–ankle pulse wave velocity
BMI	Body mass index
cfPWV	Carotid–femoral pulse wave velocity
CI	Confidence interval
CVR	Cardiovascular risk
CVRFs	Cardiovascular risk factors
CVD	Cardiovascular disease
ECG	Electrocardiogram
IBSAL	Institute of Biomedical Research of Salamanca
IPAQ-SF	International Physical Activity Questionnaire–Short Form
IUs	International units
MEDAS	Mediterranean Diet Adherence Screener
MET	Metabolic equivalent of task
PWV	Pulse wave velocity
REDIAPP	Primary Care Research Group of Castilla y León
RICAPPS	Research Network on Chronicity, Primary Care and Health Promotion
SDoH	Social determinants of health
STROBE	Strengthening the Reporting of Observational Studies in Epidemiology
WHO	World Health Organization
