# Supplementary material for: Association Between Increased Central and Peripheral Arterial 2 Stiffness and Vitamin Intake in Healthy Adults: EVA Follow-Up 3 Study
Source: Nutrients. 2026 Feb 26;18(5):745. doi: 10.3390/nu18050745 (PMC12986404; doi:10.3390/nu18050745)
Supplement: Supplementary file 1 [file nutrients-18-00745-s001.zip › nutrients-4141561-supplementary.pdf]

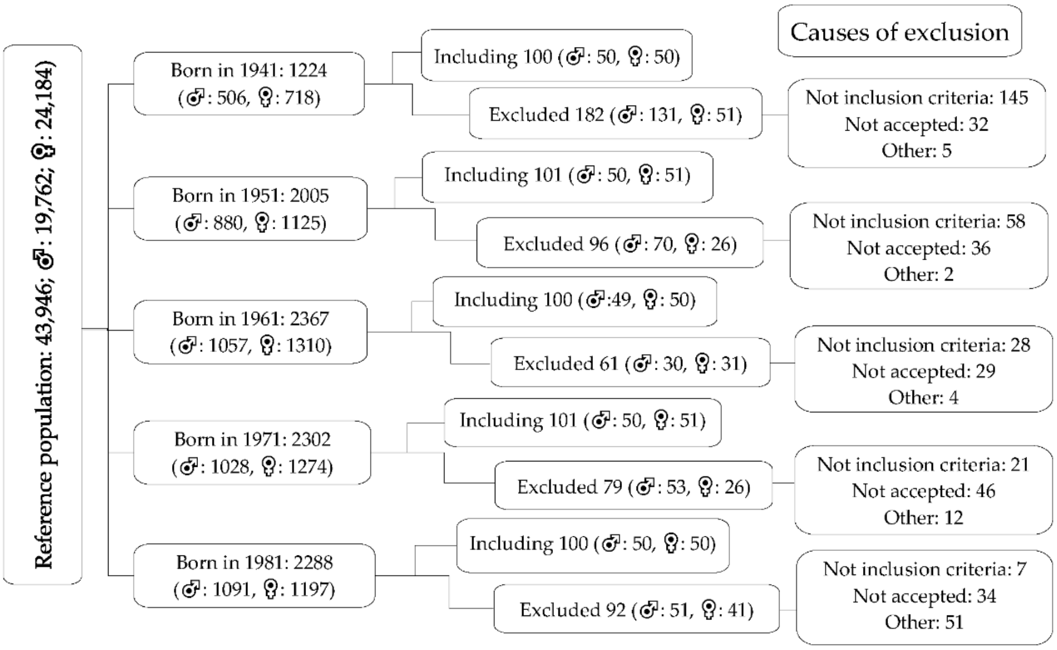

Figure S1. EVA study flowchart. It indicates the reference population by age group of the total population and by sex, the subjects included and excluded and the main causes of exclusion. 259 subjects did not meet inclusion criteria. 177 did not agree to participate in the study and 74 subjects could not be reached because they had changed their address or telephone number. The replenishment rate was 35.4 per cent and the response rate was 64.6 per cent. ♂: Males; ♀: females.

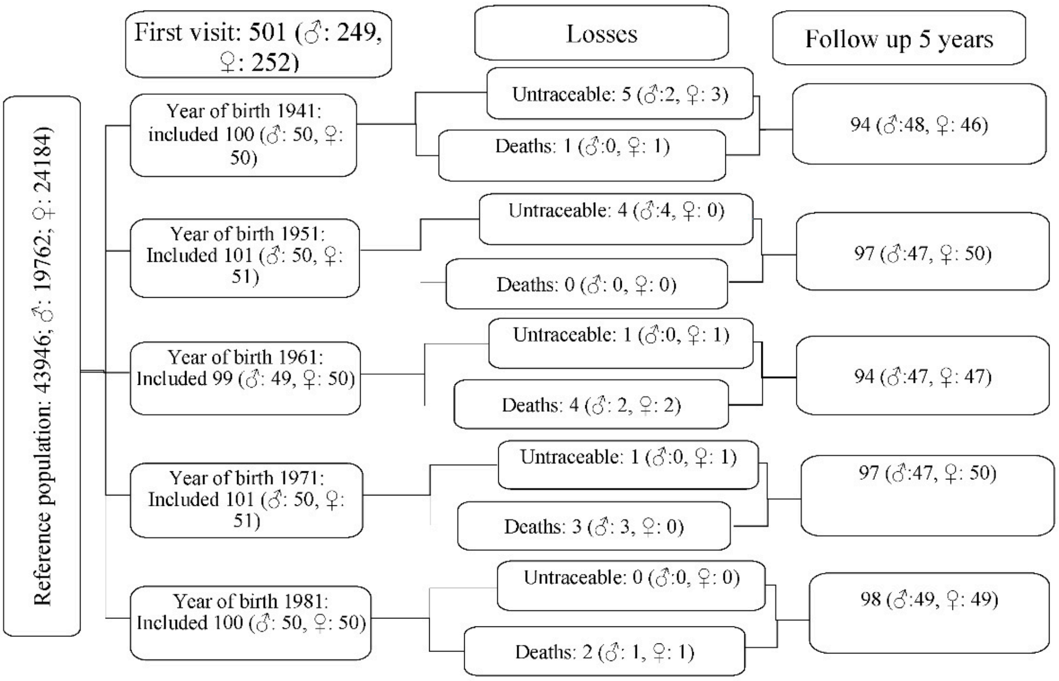

Figure S2. Flowchart of the follow-up phase of the EVA study. During the 5 years of follow-up, 10 subjects died (6♂: males; 4 ♀: females) and we were unable to contact 11 people (6 ♂: males; 5 ♀: females).

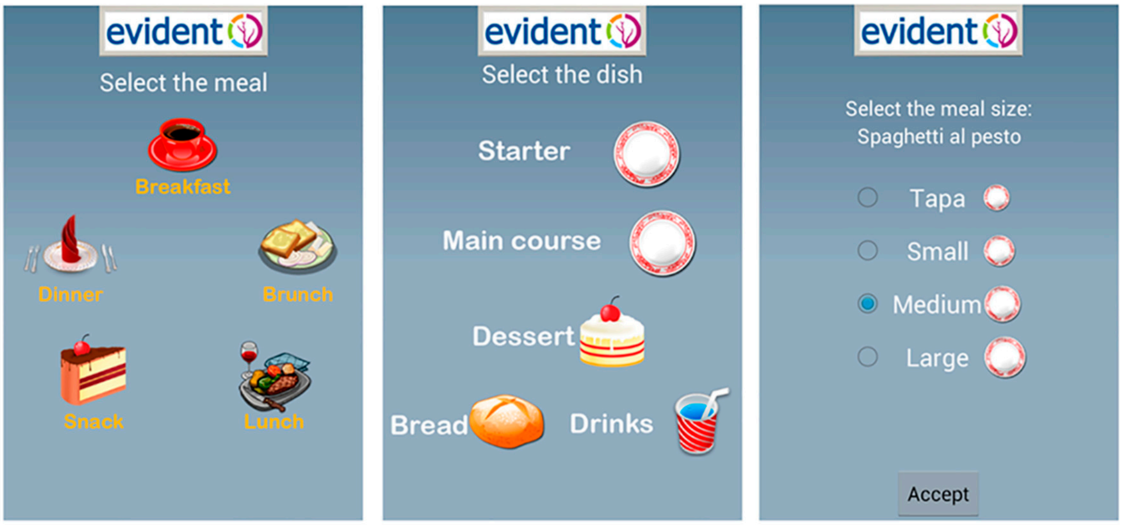

Figure S3. EVIDENT app main screen and selection of dishes.
